# Epilepsy and proxy‐reported health‐related quality of life in children and young people with non‐ambulatory cerebral palsy

**DOI:** 10.1111/dmcn.15336

**Published:** 2022-07-12

**Authors:** Adam P. Ostendorf, Erika T. Axeen, Krista Eschbach, Erin Fedak Romanowski, Lindsey A. Morgan, Paul Gross, Unni G. Narayanan, Laurie Glader, Garey Noritz, For the Cerebral Palsy Research Network, Ammar Shaikhouni, Mariah Eisner

**Affiliations:** ^1^ Department of Pediatrics Nationwide Children's Hospital and The Ohio State University College of Medicine Columbus Ohio USA; ^2^ Departments of Neurology and Pediatrics University of Virginia Virginia USA; ^3^ Children's Hospital Colorado University of Colorado Anschutz School of Medicine Aurora Colorado USA; ^4^ Department of Pediatrics, Division of Pediatric Neurology CS Mott Children's Hospital and University of Michigan Health Ann Arbor Michigan USA; ^5^ Seattle Children's Hospital and University of Washington Washington USA; ^6^ University of Utah Salt Lake City Utah USA; ^7^ University of Toronto and The Hospital for Sick Children Toronto Canada

## Abstract

**Aim:**

To assess the association between epilepsy characteristics and proxy‐reported health‐related quality of life (HRQoL) in children and young people with non‐ambulatory cerebral palsy (CP) and seizures.

**Method:**

This was a cross‐sectional study of 164 children and young people (74 females, 90 males; mean age 10 years 6 months, range 2–21 years, SD 5 years 5 months). Caregivers completed the Child Health Index of Life with Disabilities (CPCHILD) in an outpatient setting. We utilized univariable linear regression and multivariable modeling to study relationships between variables and CPCHILD scores.

**Results:**

Gross Motor Function Classification System levels were 37% IV and 63% V. Sociodemographic factors included the Child Opportunity Index (median 51, interquartile range [IQR] 25–80). A median of 2 (IQR 1–3) antiseizure medications (ASMs) were used, and days with seizures ranged from 0 (30%) to 28 (20%) days in the previous 4 weeks. Total CPCHILD scores decreased 2.3 points for each ASM (95% confidence interval [CI] –4.1 to −0.42). Compared to persons with focal epilepsy, those with generalized epilepsy had lower total CPCHILD scores (−5.7; 95% CI –11 to −0.55). Number of days with seizures was not associated with total CPCHILD scores.

**Interpretation:**

Proxy‐reported HRQoL was affected by epilepsy‐specific features in children and young people with severe CP.

**What this paper adds:**

- Health‐related quality of life (HRQoL) was lower with increasing numbers of antiseizure medications.
- Overall quality of life (QoL) scores were lower by a similar amount, independent of seizure frequency.
- HRQoL was lower in persons with recent hospital admissions for epilepsy.

## What this paper adds

The association between epilepsy characteristics and health‐related quality of life was assessed in 164 children and young people with non‐ambulatory cerebral palsy and epilepsy utilizing the Child Health Index of Life with Disabilities (CPCHILD). Total CPCHILD scores decreased across all degrees of seizure burden by a similar amount. Independently, total CPCHILD scores were lower in those taking more antiseizure medications.

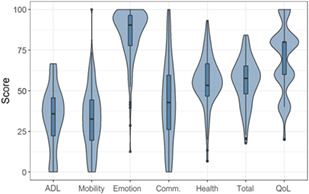

**Abbreviations**
ADL: Activities of daily living
ASM: Antiseizure medication
COI: Child Opportunity Index
CPCHILD: Child Health Index of Life with Disabilities
HRQoL: Health‐related quality of life
QoL: Quality of life
VNS: Vagus nerve stimulator

Epilepsy, a disorder of recurrent seizures, negatively impacts health‐related quality of life (HRQoL) and is a risk factor for early death.^1^ Epilepsy occurs in 30% to 50% of individuals with cerebral palsy (CP), a movement disorder resulting from non‐progressive injury to the developing brain with a high incidence of comorbid epilepsy.^2^ Together, epilepsy and CP impact an estimated 300 000 individuals in the US.^3^

Features of epilepsy and CP alter HRQoL in affected persons and their caregivers.^1^ Differences in HRQoL related to epilepsy vary based on perceived seizure control, duration of epilepsy, mental health, and peer support. The degree to which each characteristic impacts resultant quality of life (QoL) is debated, and most studies are in persons with typical neurodevelopment.^1^, ^4^, ^5^ Characteristics of CP, such as level of function, also impact QoL to varying degrees. Up to 35% of persons with CP are non‐ambulatory, and classified Gross Motor Function Classification System (GMFCS) levels IV or V.^6^ These individuals are reliant on caregivers for most of their activities of daily living (ADL), and CP can impact the activity and participation in affected persons and their caregivers.^7^, ^8^ The Caregivers Priorities and Child Health Index of Life with Disabilities (CPCHILD) was developed to measure caregivers' perception of the functional and health status, ease of caregiving, and overall well‐being in children with severe CP.^9^

Practice‐based research utilizing information obtained during routine clinical care is an advantageous method of efficiently collecting meaningful data.^10^ The Cerebral Palsy Research Network was founded to accelerate clinical investigation for CP and established a practice‐based clinical registry.^11^ This registry collects data regarding seizure frequency, seizure types, seizure treatments, and side effects alongside details regarding CP and comorbidities. We collated single‐center data from the Cerebral Palsy Research Network registry elements to comprehensive epilepsy data. We hypothesized CPCHILD scores would vary based on epilepsy features, sociodemographics, and CP characteristics.

## METHOD

This cross‐sectional study was conducted at Nationwide Children's Hospital, Columbus, Ohio, between June 2021 and October 2021. Data were collected at the time of an in‐person or virtual visit to a neurology clinic. Children and young people aged between 2 years and 21 years were enrolled if they had been diagnosed with CP with a GMFCS level IV or V as well as epilepsy. A wavier of consent was granted by Nationwide Children's Hospital Institutional Review Board.

Provider‐entered data were obtained during the clinical visit utilizing data elements embedded in forms built for the Cerebral Palsy Research Network clinical registry^12^ and an epilepsy‐specific form utilized by Nationwide Children's Hospital within the Epic electronic health record (Epic Systems Corp, Verona, WI, USA). Epilepsy type was determined by the neurology provider caring for the patient based on seizure semiology, electroencephalogram findings, and other clinical features according to the standard terminology.^13^ Providers were blinded to CPCHILD scores at time of data entry.

The Child Opportunity Index (COI) was developed and validated as an instrument to assess the potential influence of environmental conditions on child health.^14^ It consists of normalized scores from 0 to 100, with higher scores representing greater neighborhood resources which may impact health and QoL. For each child, we used the listed address in the electronic health record to locate their census tract using the Census Bureau's application programming interface (https://www.census.gov/data/developers). Then, we utilized an application programming interface to retrieve the most recent nationally normalized COI scores based on each participant's US census tract and accessed the web‐based interface for addresses that did not return a census tract.^15^ A social determinants of health score was calculated using data from screening questions for four social needs: housing, utilities, transportation, and food. These questions and methods have been previously described, and 1 point was given for each affirmative answer, summated for a total possible score of 8 indicating the greatest acute social needs.^16^

Proxy‐reported data were directly collected utilizing the electronic health record. An intake survey containing several data elements was presented to all caregivers, and an electronic version of the CPCHILD was presented to a subset if they responded that the affected person was not able to walk. The CPCHILD has five domains focused on ADL, mobility, emotion, communication, and health, and an overall QoL score is also provided.^9^ Per the scoring methodology, we removed missing values from score calculation, provided more than 50% of items were completed for each domain.

Data were collated utilizing a clinical registry within the electronic health record and exported to as a csv file via customized report. Variable distributions were visualized and quality‐checked with bar and violin plots. Data were summarized using frequency (percent) for categorical variables and median (interquartile range [IQR]) for continuous variables. While the number of days with seizures and side effects in the last 28 days were collected at a more granular level (1 day, 2–3 days, 4–14 days, 15–27 days, all 28 days, uncertain), aggregated categories of no days, some days (1, 2, 3, 4, 5, 6, 7, 8, 9, 10, 11, 12, 13, 14, 15, 16, 17, 18, 19, 20, 21, 22, 23, 24, 25, 26, 27), all 28 days, or uncertain were used for modeling because of small numbers in several categories. A composite variable was created using information about vagus nerve stimulation (VNS) and epilepsy surgery data, grouping participants into having had no surgery for epilepsy, VNS, or intracranial epilepsy surgeries (‘non‐VNS surgery’).

Univariable linear regression models were used to evaluate associations between each CPCHILD subscore and demographic and clinical variables. A combination of expert opinion and stepwise selection was used to construct multivariable models for each subscore, total CPCHILD, and overall QoL. A set of clinically interesting independent variables was included in models, regardless of statistical significance. These included the social determinants of health score, COI, GMFCS, presence of feeding tube, and history of admissions for seizures in the previous 12 months. Remaining independent variables were chosen through backward stepwise selection based on the Akaike information criterion. Multivariable models were developed using complete cases and checked for collinearity among independent variables using the variance inflation factor.^17^ Estimates were presented with 95% confidence intervals. Statistical tests were conducted using two‐sided *p‐*values and considered significant when the *p*‐value was less than 0.05. All statistical analyses were performed in R version 4.0 (R Core Team, Vienna, Austria). STROBE reporting guidelines were followed.

## RESULTS

Participants included 164 children and young people with epilepsy and non‐ambulatory CP (74 females, 90 males; mean age 10y 6mo, SD 5 years 5 months; median age 10y, IQR 6y–15y), 63% of whom were in GMFCS level V. Their characteristics are summarized in Table 1. The overall epilepsy intake survey response rate was 82%, and the rate of missingness for each included variable was less than 5%. Importantly, the COI median of 51 (IQR 25–80) was consistent with a normally distributed sample representative of a broad range of socioeconomic status. Most patient families lacked severe acute sociodemographic stressors, as measured by the social determinants of health score. Epilepsy was drug‐resistant (defined as continued seizures despite adequate trials of ≥2 antiseizure medications [ASMs]) in 62%, and 21% of persons had been admitted for epilepsy in the past 12 months. Treatment characteristics included a median of 2 (IQR 1–3) ASMs, and 21% had a history of surgery for epilepsy (15% VNS and 6% non‐VNS surgery).

**Table 1:** Patient demographics and characteristics

Characteristic	*n*=164
Age (y)	10 (6–15)
Female sex	74 (45)
Ethnic group	
White	119 (73)
Black	30 (18)
Other	15 (9.1)
Public insurance coverage	99 (60)
Social Determinants Score	2 (1–3)
Child Opportunity Index	51 (25–80)
GMFCS level V	104 (63)
Feeding tube present	97 (59)
Epilepsy type	
Focal	88 (54)
Generalized	42 (26)
Combined focal and generalized	32 (20)
Drug‐resistant epilepsy	101 (62)
First seizure age <1y of age	97 (60)
Epilepsy duration (y)	8 (5–13)
Epilepsy surgery	34 (21)
Epilepsy surgery and type	
No surgery	130 (79)
Non‐VNS surgery	10 (6.1)
VNS	24 (15)
Family history of epilepsy	28 (17)
Number of ASMs	2 (1–3)
Seizure days in last 28 days	
No days	50 (30)
1d	9 (5.5)
2–3d	16 (9.8)
4–14d	24 (15)
15–27d	18 (11)
All 28d	33 (20)
Uncertain	14 (8.5)
Side effect days in last 28d	
No days	101 (62)
1d	2 (1.2)
2–3d	4 (2.4)
4–14d	3 (1.8)
15–27d	4 (2.4)
All 28d	14 (8.5)
Uncertain	36 (22)
Any admissions for epilepsy, previous 12mo	35 (21)

Statistics presented are median (IQR) or *n* (%).

Abbreviations: ASM, antiseizure medicine; GMFCS, Gross Motor Function Classification System; VNS, vagus nerve stimulator.

**Figure 1:**
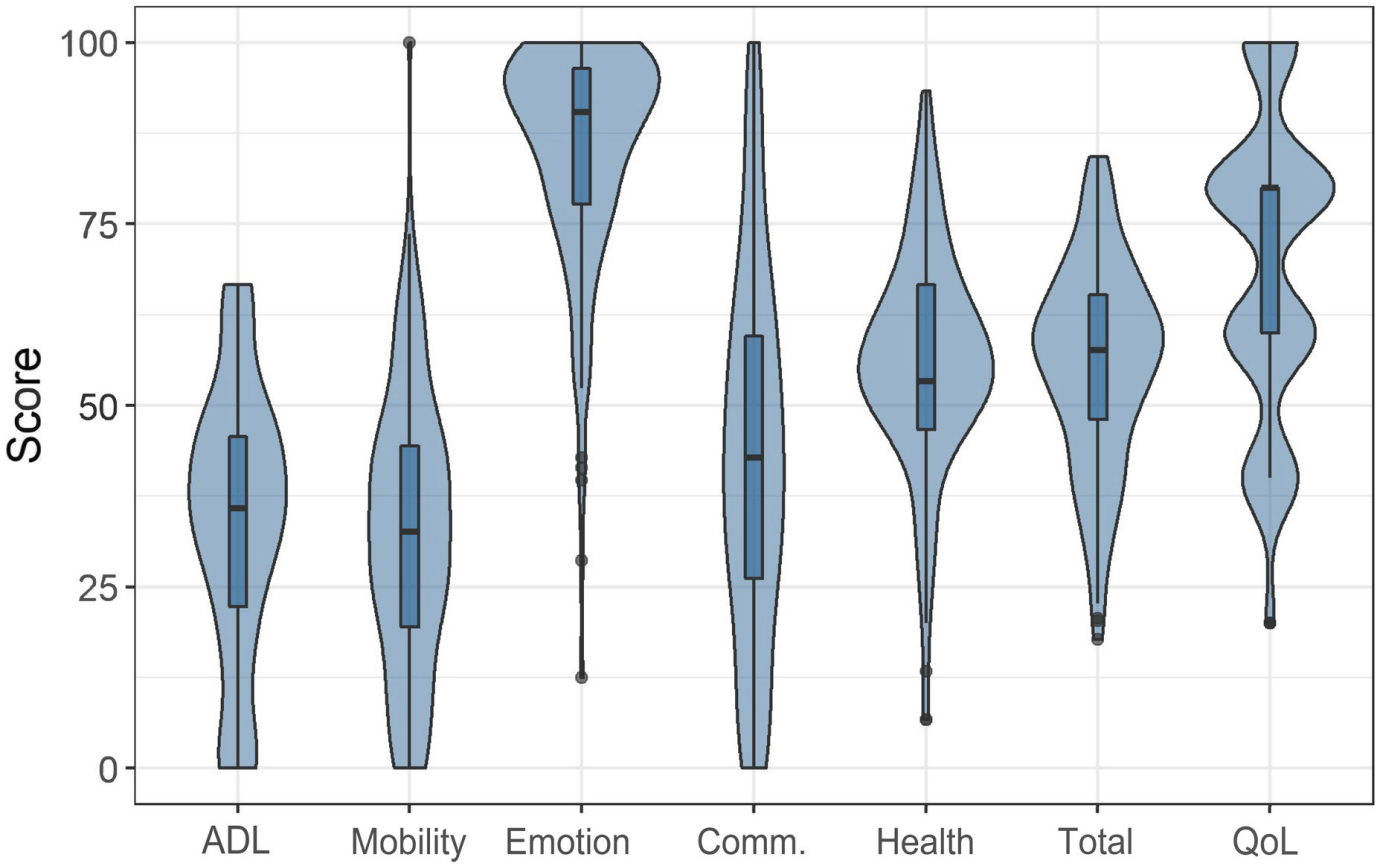
Median Child Health Index of Life with Disabilities scores and distributions across domains, total score, and overall quality of life (QoL) displayed in violin plots. ADL, activities of daily living.

Median (IQR) CPCHILD scores (Figure 1) were as follows: total CPCHILD 58 (48–65); ADL 36 (22–46); mobility 33 (19–44); comfort, emotion, and behavior (‘emotion’) 90 (78–96); communication and social interaction (‘communication’) 43 (26–60); health 53 (47–67); and overall QoL score 80 (60–80). Several individual patient characteristics were significantly associated with overall QoL score, total score, and domain scores in the final multivariable models (Table 2 and Table 3). Estimated mean overall QoL scores were associated with a decrease of 3.3 for each ASM (95% CI –6.2 to −0.35). Independently, any number of seizures in the past 28 days was associated with a decrease in the overall QoL. Specifically, the estimated mean decrease in overall QoL score was similar for 1 to 27 days (−10; 95% CI –19 to −2.0), all 28 days (−17; 95% CI –27 to −6.0), and those who were uncertain (−15; 95% CI –27 to −3.1), where uncertain likely reflected difficulty distinguishing between more granular category choices (i.e. every 2–3 days vs every 4–14 days). QoL was higher in individuals with combined focal and generalized epilepsy (vs focal only), or whose ethnicity was neither White nor Black.

**Table 2:** Estimated regression coefficients for variables included in multivariable models for overall QoL, total CPCHILD score, and health domain score in CPCHILD

	Overall QoL			Total CPCHILD score			CPCHILD health domain		
Characteristic	Coefficient	95% CI	*p*	Coefficient	95% CI	*p*	Coefficient	95% CI	*p*
Sex, male vs female	–	–	–	6.1	1.8, 10	**0.006**	4.8	0.23, 9.4	**0.040**
Ethnic group, vs White									
Black	−3.6	−12, 5.0	0.4	−1.7	−7.7, 4.2	0.6	−3.3	−9.9, 3.3	0.3
Other	12	0.83, 23	**0.035**	6.9	−0.66, 14	0.074	7.2	−0.94, 15	0.082
Insurance, public vs commercial	–	–	–	−4.0	−8.9, 1.0	0.11	–	–	–
Social Determinant Score	−0.91	−2.8, 0.94	0.3	−0.57	−1.9, 0.72	0.4	−0.68	−2.0, 0.68	0.3
COI, 10 point change	−0.71	−1.8, 0.38	0.2	−0.51	−1.3, 0.29	0.2	−0.83	−1.6, −0.01	**0.046**
GMFCS level, V vs IV	−1.7	−9.3, 5.9	0.7	−7.0	−12, −1.9	**0.007**	−7.0	−13, −1.2	**0.018**
Feeding tube	0.40	−6.8, 7.6	>0.9	2.5	−2.4, 7.3	0.3	−2.0	−7.3, 3.3	0.5
Epilepsy type, vs focal									
Generalized	2.2	−5.3, 9.7	0.6	−5.7	−11, −0.55	**0.030**	–	–	–
Combined focal and generalized	13	4.4, 22	**0.004**	−0.36	−6.3, 5.6	>0.9	–	–	–
First seizure ≥1y of age	−4.7	−11, 2.0	0.2	–	–	–	−6.6	−12, −1.6	**0.010**
Epilepsy duration (years)	–	–	–	–	–	–	–	–	–
Epilepsy surgery and type, vs no surgery	–	–	–	–	–	–	–	–	–
Non‐VNS surgery	–	–	–	–	–	–	–	–	–
VNS	–	–	–	–	–	–	–	–	–
Family history of epilepsy	–	–	–	–	–	–	–	–	–
Number of ASMs	−3.3	−6.2, −0.35	**0.028**	−2.3	−4.1, −0.42	**0.016**	−1.5	−3.5, 0.45	0.13
Seizure days in last 28 days, vs no days									
Some days (1–27)	−10	−19, −2.0	**0.015**	–	–	–	–	–	–
All 28d	−17	−27, −6.0	**0.002**	–	–	–	–	–	–
Uncertain	−15	−27, −3.1	**0.014**	–	–	–	–	–	–
Side effect days in last 28, vs no days									
Some days (1–27)	–	–	–	–	–	–	–	–	–
All 28 days	–	–	–	–	–	–	–	–	–
Uncertain	–	–	–	–	–	–	–	–	–
Any admissions for epilepsy	−5.6	−13, 2.2	0.2	4.2	−1.0, 9.5	0.11	−6.2	−12, −0.51	**0.033**

Bold type indicates statistical significance. Variables evaluated but not included in the multivariable models are indicated with a dash. Abbreviations: ASM, antiseizure medicine; CI, confidence interval; COI: Child Opportunity Index; CPCHILD, Child Health Index of Life with Disabilities; GMFCS, Gross Motor Function Classification Scale; QoL, quality of life; VNS, vagus nerve stimulator.

**Table 3:** Estimated regression coefficients for variables included in multivariable models for communication, emotion, mobility, and ADL domains in CPCHILD

	Communication domain			Emotion domain			Mobility domain			ADL domain		
Characteristic	Coefficient	95% CI	*p*	Coefficient	95% CI	*p*	Coefficient	95% CI	*p*	Coefficient	95% CI	*p*
Sex, male vs female	–	–	–	–	–	–	–	–	–	11	5.1, 16	**<0.001**
Ethnic group, vs White												
Black	–	–	–	–	–	–	−6.9	−14, 0.73	0.076	−4.7	−13, 3.1	0.2
Other	–	–	–	–	–	–	9.8	0.32, 19	**0.043**	10	0.43, 20	**0.041**
Insurance, public vs commercial	–	–	–	−7.1	−13, −1.6	**0.012**	–	–	–	–	–	–
Social History Score	−0.68	−2.9, 1.5	0.5	−0.40	−1.8, 1.0	0.6	−1.4	−3.1, 0.18	0.082	−0.23	−1.9, 1.4	0.8
COI, 10 point change	0.56	−0.74, 1.9	0.4	−1.2	−2.1, −0.36	**0.006**	0.49	−0.47, 1.4	0.3	−0.22	−1.2, 0.77	0.7
GMFCS level, V vs IV	−20	−29, −12	**<0.001**	−5.2	−11, 0.56	0.077	−11	−17, −4.0	**0.002**	−1.7	−8.4, 4.9	0.6
Feeding tube	3.2	−5.7, 12	0.5	4.4	−1.1, 9.8	0.12	−1.9	−8.0, 4.2	0.5	2.1	−4.3, 8.5	0.5
Epilepsy type, vs focal												
Generalized	–	–	–	–	–	–	−7.5	−14, −0.87	**0.027**	−6.7	−14, 0.11	0.054
Combined focal and generalized	–	–	–	–	–	–	−5.5	−13, 2.1	0.2	−3.7	−11, 4.1	0.4
First seizure ≥1y of age	–	–	–	–	–	–	–	–	–	–	–	–
Epilepsy duration (y)	–	–	–	–	–	–	–	–	–	–	–	–
Epilepsy surgery and type, vs no surgery	–	–	–	–	–	–	–	–	–	–	–	–
Non‐VNS surgery	–	–	–	–	–	–	–	–	–	–	–	–
VNS	–	–	–	–	–	–	–	–	–	–	–	–
Family history of epilepsy	–	–	–	–	–	–	5.4	−2.0, 13	0.15	–	–	–
Number of ASMs	–	–	–	−2.3	−4.2, −0.29	**0.025**	−2.2	−4.5, 0.19	0.071	−2.0	−4.4, 0.45	0.11
Seizure days in last 28 days, vs no days												
Some days (1–27)	−12	−21, −2.8	**0.011**	–	–	–	–	–	–	–	–	–
All 28d	−14	−26, −2.3	**0.019**	–	–	–	–	–	–	–	–	–
Uncertain	−15	−29, −0.83	**0.038**	–	–	–	–	–	–	–	–	–
Side effect days in last 28, vs no days												
Some days (1–27)	–	–	–	–	–	–	–	–	–	–	–	–
All 28d	–	–	–	–	–	–	–	–	–	–	–	–
Uncertain	–	–	–	–	–	–	–	–	–	–	–	–
Any admissions for epilepsy	7.5	−1.9, 17	0.12	−1.1	−6.9, 4.8	0.7	5.1	−1.5, 12	0.13	1.5	−5.5, 8.4	0.7

Bold type indicates statistical significance. Variables evaluated but not included in the multivariable models are indicated with a dash. Abbreviations: ADL, activities of daily living; ASM, antiseizure medicine; CI, confidence interval; COI, Child Opportunity Index; GMFCS, Gross Motor Function Classification Scale; VNS, vagus nerve stimulator.

Total CPCHILD scores were most negatively influenced by GMFCS level V (−7; 95% CI –12 to −1.9), female sex (−6.1; 95% CI –10 to −1.8), and having generalized compared to focal epilepsy (−5.7; 95% CI –11 to −0.55). Importantly, an increasing number of ASMs was also negatively associated with total score (−2.3; 95% CI –4.1 to −0.42).

An increasing number of ASMs negatively impacted emotion scores, as did having public insurance and increasing COI. Health scores were also negatively impacted by increasing COI, in addition to GMFCS level V, any epilepsy admissions in the previous 12 months, female sex, and onset of seizures after the first year of life. Communication scores were adversely impacted by both GMFCS level V and having had any number of reported seizures in the previous 28 days. The ADL domain was least impacted by seizure‐related characteristics. Males and persons who were neither White nor Black had higher scores in the multivariable models.

## DISCUSSION

This is the first study of proxy‐reported HRQoL in children, adolescents, and young people with non‐ambulatory CP and epilepsy utilizing a specific measure for this high‐risk population and detailed epilepsy data. Notably, we identified an association between increasing numbers of ASMs and lower total CPCHILD score. Treatment with more than two concurrent ASMs is unlikely to provide long‐term seizure control or other benefit to persons with drug‐resistant epilepsy, yet ASM overtreatment exists.^18^, ^19^ Increasing polypharmacy has been associated with lower HRQoL in other populations,^20^ but our study of this group is novel. Importantly, we identified this association on multivariable analysis, isolating the effect from ASM number from those related to seizure frequency or epilepsy‐related hospital admissions.

Individuals with any days affected by seizures in the last 4 weeks had lower overall QoL scores of more than 10 points. The association appeared similar whether a patient had 1 day or 28 days with seizures in the previous 4 weeks, which is consistent with previous studies of HRQoL in individuals with epilepsy demonstrating a significant sustained improvement in HRQoL only with at least 12 months of seizure freedom.^21^ The association between HRQoL and seizure control in non‐ambulatory individuals with CP was previously explored, but without other important epilepsy‐related characteristics. In a group of 66 children, Elema et al. reported individuals without epilepsy or with less than one seizure per month had a higher CPCHILD overall QoL score compared to those with one or more per month.^3^ Individuals with and without epilepsy were combined for analysis, despite previous data demonstrating those with epilepsy and one seizure per year have lower QoL compared to those who are seizure‐free.^22^ Arnaud et al. reported children across all GMFCS levels with CP who had seizures more than one time per month had lower proxy‐reported social domain QoL using a different instrument.^23^ Others found negative effects from seizures on QoL measured using the Child Health Questionnaire in smaller subsets of individuals with severe CP and epilepsy.^24^, ^25^ Therefore, our report is novel in specifically examining the impact of seizure frequency alongside other epilepsy characteristics in a large group of individuals with severe CP.

Other epilepsy‐related characteristics influenced CPCHILD scores. Total CPCHILD score was lower in those with generalized epilepsy. This may be related to CP and epilepsy etiology (i.e. genetic vs structural) and resultant functional differences, or seizure type, as generalized seizures have been associated with worse HRQoL.^26^ Any hospital admissions for epilepsy were negatively associated with health domain scores (−6.2; 95% CI –12 to −0.5), similar to previous reports for the health domain, but with a lower estimated impact.^3^ Age of seizure onset over 1 year was inversely associated with health domain scores (−6.6; 95% CI –12 to −1.7), which is consistent with previous reports of lower QoL in adults.^27^

Our study was also innovative in utilizing COI, a multidimensional, population‐level surveillance tool that incorporates attributes of neighborhood conditions that may impact child health.^14^ We identified an inverse correlation between COI and the health and emotion domain scores. This appears counterintuitive at first glance because of the more commonly identified link between lower socioeconomic status and lower QoL.^28^ However, previous authors also found associations between higher socioeconomic status and lower proxy‐reported QoL in children with CP.^3^, ^23^ Arnaud and colleagues postulated that this results from discordance between greater expectations of families with higher socioeconomic status and the reality of caring for a child with severe CP,^23^ although this hypothesis has yet to be explored.

Several findings related to CP and sociodemographics were notable. First, those with public health insurance had lower emotion domain scores. In the US, public health insurance is often associated with decreased access to health care services and having more high‐risk social determinants of health.^29^ The lower emotion domain score could reflect the impact of these co‐occurring stressors, although insurance type did not significantly impact the total CPCHILD score. Second, scores for overall QoL, the mobility domain, and the ADL domain were higher for persons who were neither White nor Black. Differences in HRQoL related to ethnicity in persons with epilepsy or CP in the US have not been clearly reported and require further study. Similar to a previous study utilizing the CPCHILD, we found a negative association between GMFCS level V and mobility, communication, health, and total scores.^3^

This study utilized practice‐based data collection leveraging numerous elements of the Cerebral Palsy Research Network clinical registry, allowing for a large sample of consecutive patients with more than 90% data completeness. However, we did not capture other data elements of potential interest, such as specific caregiver education level and family structure.^3^ We mitigated some of these concerns by utilizing methods used elsewhere, such as social determinants of health score, COI, and primary insurance payer. This report is from a single large academic center and limited by lack of representation across specific demographics, such as individuals who were neither White nor Black. Future studies leveraging multicenter practice‐based registries may validate the generalizability of our findings.^30^

Persons with non‐ambulatory CP and epilepsy are at risk for having reduced proxy‐reported HRQoL. Prospective interventional studies are needed to test if caregivers' perception of the ease of care, comfort, health, and well‐being of the child can be improved, potentially by reducing the degree of ASM polypharmacy or using treatments more likely to result in seizure freedom, such as epilepsy surgery.

## Data Availability

The data that support the findings of this study are available on request from the corresponding author. The data are not publicly available due to privacy or ethical restrictions.

## References

[1] Ostendorf AP , Gedela S . Effect of Epilepsy on Families, Communities, and Society. Semin Pediatr Neurol 2017; 24: 340–7. 29249514 10.1016/j.spen.2017.10.007

[2] Christensen D , Van Naarden Braun K , Doernberg NS , et al. Prevalence of cerebral palsy, co‐occurring autism spectrum disorders, and motor functioning ‐ Autism and Developmental Disabilities Monitoring Network, USA, 2008. Dev Med Child Neurol 2014; 56: 59–65. 24117446 10.1111/dmcn.12268 PMC4351771

[3] Elema A , Zalmstra TAL , Boonstra AM , Narayanan UG , Reinders‐Messelink HA , V D Putten AAJ. Pain and hospital admissions are important factors associated with quality of life in nonambulatory children. Acta Paediatr 2016; 105: e419–425. 27250697 10.1111/apa.13493

[4] Fayed N , Davis AM , Streiner DL , et al. Children's perspective of quality of life in epilepsy. Neurology 2015; 84: 1830–7. 25841031 10.1212/WNL.0000000000001536 PMC4433469

[5] Ronen GM , Rosenbaum PL , Boyle MH , Streiner DL . Patient‐reported quality of life and biopsychosocial health outcomes in pediatric epilepsy: An update for healthcare providers. Epilepsy Behav 2018; 86: 19–24. 30036765 10.1016/j.yebeh.2018.05.009

[6] Palisano R , Rosenbaum P , Walter S , Russell D , Wood E , Galuppi B . Development and reliability of a system to classify gross motor function in children with cerebral palsy. Dev Med Child Neurol 1997; 39: 214–23. 9183258 10.1111/j.1469-8749.1997.tb07414.x

[7] Hallum A , Krumboltz JD . Parents caring for young adults with severe physical disabilities: psychological issues. Dev Med Child Neurol 1993; 35: 24–32. 8449377 10.1111/j.1469-8749.1993.tb11548.x

[8] Gutierrez‐Angel AM , Martinez‐Juarez IE , Hernandez‐Vanegas LE , Crail‐Melendez D . Quality of life and level of burden in primary caregivers of patients with epilepsy: Effect of neuropsychiatric comorbidity. Epilepsy Behav 2018; 81: 12–7. 29455081 10.1016/j.yebeh.2018.01.034

[9] Narayanan UG , Fehlings D , Weir S , Knights S , Kiran S , Campbell K . Initial development and validation of the Caregiver Priorities and Child Health Index of Life with Disabilities (CPCHILD). Dev Med Child Neurol 2006; 48: 804–12. 16978459 10.1017/S0012162206001745

[10] Narayanan J , Dobrin S , Choi J , et al. Structured clinical documentation in the electronic medical record to improve quality and to support practice‐based research in epilepsy. Epilepsia 2017; 58: 68–76. 10.1111/epi.13607 PMC5245120 27864833

[11] Hurvitz EA , Gross PH , Gannotti ME , Bailes AF , Horn SD . Registry‐based Research in Cerebral Palsy: The Cerebral Palsy Research Network. Phys Med Rehabil Clin N Am 2020; 31: 185–94. 31760991 10.1016/j.pmr.2019.09.005

[12] Gross P , Gannotti M , Bailes A , et al. Cerebral Palsy Research Network Clinical Registry: Methodology and Baseline Report. Arch Rehabil Res Clin Transl 2020; 2: 100054. 33543081 10.1016/j.arrct.2020.100054 PMC7853390

[13] Scheffer IE , Berkovic S , Capovilla G , et al. ILAE classification of the epilepsies: Position paper of the ILAE Commission for Classification and Terminology. Epilepsia 2017; 58: 512–21. 28276062 10.1111/epi.13709 PMC5386840

[14] Acevedo‐Garcia D , McArdle N , Hardy EF , et al. The child opportunity index: improving collaboration between community development and public health. Health Aff (Millwood) 2014; 33: 1948–57. 25367989 10.1377/hlthaff.2014.0679

[15] diversitydatakids.org. Child Opportunity Index 2.0 database. https://data.diversitydatakids.org/dataset/coi20‐child‐opportunity‐index–2–0‐database?_external=True (accessed 31 Oct 2021).

[16] Hardy R , Boch S , Keedy H , Chisolm D . Social Determinants of Health Needs and Pediatric Health Care Use. J Pediatr 2021; 238: 275–281.e1. 34329688 10.1016/j.jpeds.2021.07.056

[17] Marquardt D. Generalized Inverses, Ridge Regression, Biased Linear Estimation, and Nonlinear Estimation. Technometrics 1970; 12: 591–612.

[18] Brodie MJ , Barry SJE , Bamagous GA , Norrie JD , Kwan P . Patterns of treatment response in newly diagnosed epilepsy. Neurology 2012; 78: 1548–54. 22573629 10.1212/WNL.0b013e3182563b19 PMC3348850

[19] Perucca E , Kwan P . Overtreatment in epilepsy: how it occurs and how it can be avoided. CNS Drugs 2005; 19: 897–908. 16268662 10.2165/00023210-200519110-00001

[20] Alexander HB , Broshek DK , Quigg M . Quality of life in adults with epilepsy is associated with anticonvulsant polypharmacy independent of seizure status. Epilepsy Behav 2018; 78: 96–9. 29179106 10.1016/j.yebeh.2017.11.006

[21] Kwan P , Arzimanoglou A , Berg AT , et al. Definition of drug resistant epilepsy: consensus proposal by the ad hoc Task Force of the ILAE Commission on Therapeutic Strategies. Epilepsia 2010; 51: 1069–77. 19889013 10.1111/j.1528-1167.2009.02397.x

[22] Dwivedi R , Ramanujam B , Chandra PS , et al. Surgery for Drug‐Resistant Epilepsy in Children. The New England Journal of Medicine 2017; 377: 1639–47. 29069568 10.1056/NEJMoa1615335

[23] Arnaud C , White‐Koning M , Michelsen SI , et al. Parent‐reported quality of life of children with cerebral palsy in Europe. Pediatrics 2008; 121: 54–64. 18166557 10.1542/peds.2007-0854

[24] Vargus‐Adams J. Health‐related quality of life in childhood cerebral palsy. Arch Phys Med Rehabil 2005; 86: 940–5. 15895340 10.1016/j.apmr.2004.10.036

[25] Wake M , Salmon L , Reddihough D . Health status of Australian children with mild to severe cerebral palsy: cross‐sectional survey using the Child Health Questionnaire. Dev Med Child Neurol 2003; 45: 194–9. 12613777 10.1017/s0012162203000379

[26] Baker GA , Gagnon D , McNulty P . The relationship between seizure frequency, seizure type and quality of life: findings from three European countries. Epilepsy Res 1998; 30: 231–40. 9657650 10.1016/s0920-1211(98)00010-2

[27] Szaflarski M , Meckler JM , Privitera MD , Szaflarski JP . Quality of life in medication‐resistant epilepsy: the effects of patient's age, age at seizure onset, and disease duration. Epilepsy Behav 2006; 8: 547–51. 16483851 10.1016/j.yebeh.2006.01.001

[28] Didsbury MS , Kim S , Medway MM , et al. Socio‐economic status and quality of life in children with chronic disease: A systematic review. J Paediatr Child Health 2016; 52: 1062–9. 27988995 10.1111/jpc.13407

[29] Access to Health Services. Healthy People 2020. 2021. https://www.healthypeople.gov/2020/leading‐health‐indicators/2020‐lhi‐topics/Access‐to‐Health‐Services (accessed 22 Dec 2021).

[30] Lungu C , Hirtz D , Damiano D , Gross P , Mink JW . Report of a workshop on research gaps in the treatment of cerebral palsy. Neurology 2016; 87: 1293–8. 27558377 10.1212/WNL.0000000000003116 PMC5035982

